# Double-layered collagen graft to the radial forearm free flap donor sites without skin graft

**DOI:** 10.1186/s40902-015-0046-9

**Published:** 2015-12-01

**Authors:** Tae-Jun Park, Hong-Joon Kim, Kang-Min Ahn

**Affiliations:** Department of Oral and Maxillofacial Surgery, College of Medicine, University of Ulsan, Seoul Asan Medical Center, 88 Olympic-ro 43-gil, Songpa-gu, 138-736 Seoul South Korea

## Abstract

**Background:**

Radial forearm free flap is the most reliable flap for intraoral soft tissue reconstruction after cancer ablation surgery. However, unesthetic scar of the donor site and the need for a second donor site for skin graft are major disadvantages of the forearm flap. The purpose of this study was to report the clinical results of double-layered collagen graft to the donor site of the forearm free flap without skin graft.

**Methods:**

Twenty-two consecutive patients who underwent oral cancer ablation and forearm reconstruction between April 2010 and November 2013 were included in this study. Male to female ratio was 12:10, and average age was 61.0 years old (27–84). Double-layered collagen was grafted to the donor site of the forearm free flap and healed for secondary intention. Upper silicone had been trimmed at the periphery during secondary intention, and dry dressing was used. Postoperative scar healing and esthetic results and function were evaluated.

**Results:**

An average follow-up period was 34.9 months. The scar area was decreased to 63.9 % in average. The complete healing was obtained between 1.5 and 3 months according to the defect size. There was no functional defect or impairment 3 months after operation. All patients were satisfied with the esthetic results. Three patients died of recurred cancer.

**Conclusions:**

Double-layered collagen graft was successfully performed in this study. Without the thigh skin graft, patients had experienced less painful postoperative healing periods and discomfort.

## Background

Since the early 1980s, the radial forearm free flap (RFFF) has been used for head and neck reconstruction due to its thin, hairless skin, pliable soft tissue, consistent anatomy, and long vascular pedicle [1–3]. However, donor site morbidities have been reported such as poor wound healing, tendon exposure, skin graft failure, and poor esthetic results. Although the long-term morbidity at the radial donor site is often relatively minor, and to most oncological patients, the morbidities may be subsidiary issues, prolonged wound healing causes undesirable inconveniences and poor esthetic results [4]. The defects of donor site are generally large for primary closure, which needs skin graft for dressing. Acceptable managements of such large defects are full-thickness skin graft (FTSG) and split-thickness skin grafts (STSG) [5]. For healing of the forearm donor site, FTSG may be prior to STSG due to its sufficient bulk and thickness. As a result of a sufficient amount of dermis, superior esthetic and functional outcomes in FTSG are suggested by promoted wound healing and the decrease of tendon adhesion [6]. However, because of severe functional and esthetic morbidities of the second donor site, FTSGs are able to adopt only in limited cases [7]. With STSG, compared to FTSG, the healing period of second donor site is shorter and the morbidity of second donor site is smaller [8]. But, donor site management with STSG also has complications including partial or total necrosis of the skin and poor wound healing. These complications are caused as a result of deficient bulk, and they may lead to restriction of muscle movement, tendon exposure, and widespread wound scarring. In addition, the STSG at the radial forearm donor site may result in esthetically poor wound contour with irregular wound texture [5]. In general, the STSGs were harvested from the thigh, and therefore, the morbidities include remained scar, pain, movement disorders, and difficulties in follow-up dressings [6]. Wester et al. found that composite grafting of artificial dermis and ultrathin STSG showed greater patient satisfaction and functional recovery of the forearm compared with STSG alone [9]. They suggested that using artificial dermis in combination with STSG as a composite graft provides an additional bulk of tissue with the advantage of reducing tissue contracture with minimal functional morbidity [9]. The combination of artificial dermis and a STSG enables the forearm donor site to recover with a newly formed full-thickness skin [10]. However, this procedure needs another operation and has the morbidity of second donor site.

The purpose of this study was to evaluate clinical results of double-layered collagen graft technique for radial forearm donor site reconstruction without skin graft.

## Methods

Twenty-two consecutive patients between April 2010 and September 2014 were included in this study. Institutional review board from Asan Medical Center issued an exemption to this study because of the use of collected existing data in such a manner that subjects cannot be identified, directly or through identifiers linked to subjects. Male to female was 12:10 and average age was 61 years old. The age of the patients ranged from 27 to 84 years. The mean follow-up period was 34.9 months. Only one patient was revealed melanoma and the others were squamous cell carcinoma histopathologically.

**Fig. 1:**
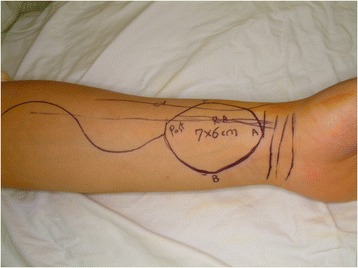
Design of the radial forearm free flap during operation

**Fig. 2:**
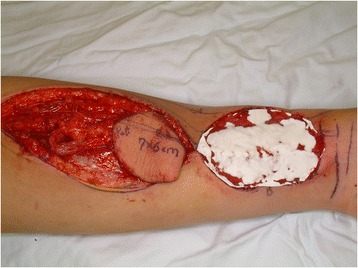
Application of MatriDerm® onto the donor site

**Fig. 3:**
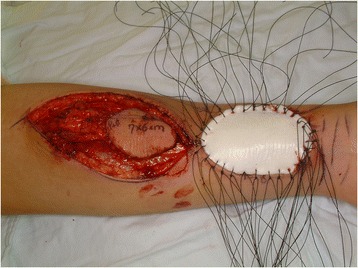
Application of Terudermis® onto the outer surface of MatriDerm®

**Fig. 4:**
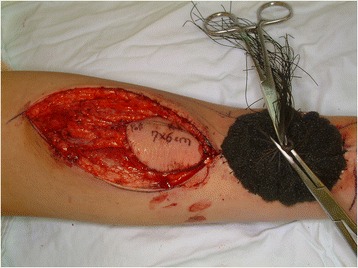
Wound coverage with tie-over dressing

RFFF was elevated by subfascial plane under general anesthesia. After the elevation of RFFF, adjacent muscles covered exposed the brachioradialis tendon. A MatriDerm® (MedSkin Solution, Billerbeck, Germany) sheet on the bottom layer and a Terudermis® (Terumo Corporation, Tokyo, Japan) sheet on the surface layer fitted to the defect site. Double-layered collagens were fixed with 2-0 silk temporary sutures, and a tie-over dressing was applied. A wrist-around splint was performed for 1 week to stabilize the graft and to minimize wound contractures (Figs. 1, 2, 3, and 4). One week after surgery, tie-over dressing was removed and a patient had the 2-0 silk stitches taken out. The silicone layer of Terudermis was trimmed at the periphery during secondary intention healing period, and dry dressing with powdered medicine was applied.

Before the elevation of RFFF, the length and width of the flap were measured, and the length multiplied by width was recorded as initial scar area. Tie-over dressing was removed a week after surgery. The scar area was measured when the patients came to the outpatient clinic for follow-up checks. The area change had been recorded, and the ratio before and after operation was calculated.

## Results

**Fig. 5:**
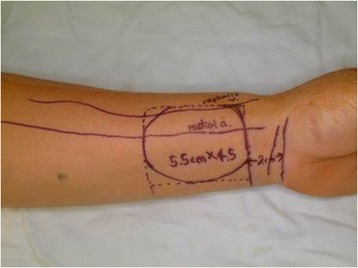
During operation (patient no. 1)

**Fig. 6:**
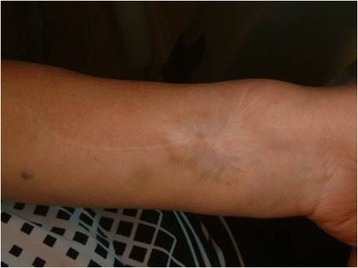
Postoperation 4 years 11 months (patient no. 1)

**Fig. 7:**
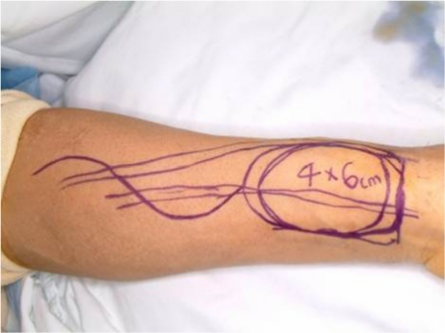
During operation (patient no. 3)

**Fig. 8:**
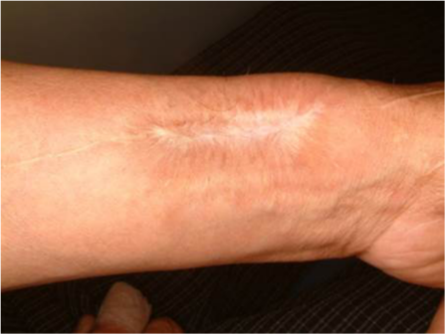
Postoperation 4 years 8 months (patient no. 3)

**Fig. 9:**
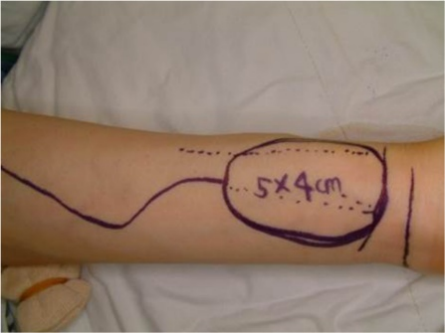
During operation (patient no. 4)

**Fig. 10:**
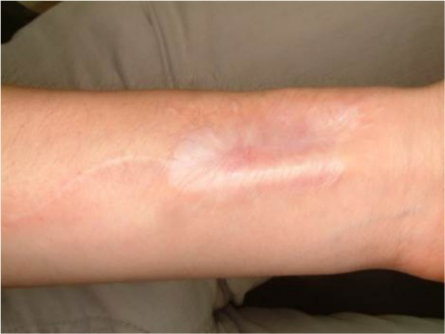
Postoperation 3 years 6 months (patient no. 4)

**Fig. 11:**
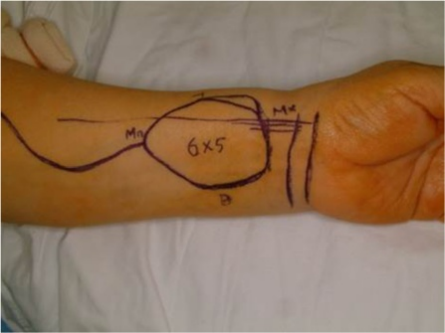
During operation (patient no. 5)

**Fig. 12:**
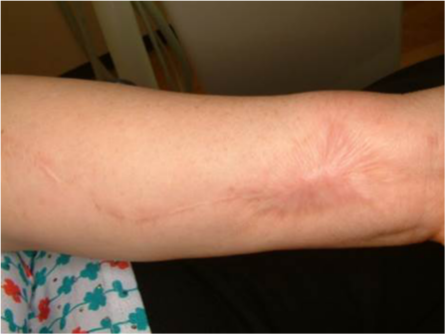
Postoperation 2 years (patient no. 5)

**Fig. 13:**
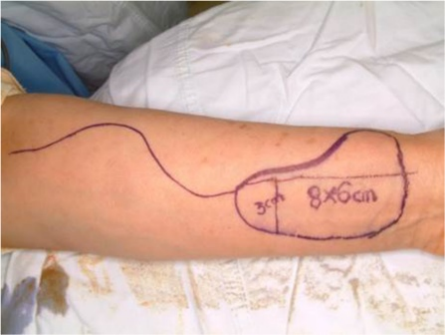
During operation (patient no. 6)

**Fig. 14:**
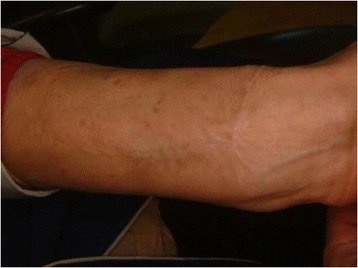
Postoperation 4 years 5 months (patient no. 6)

**Fig. 15:**
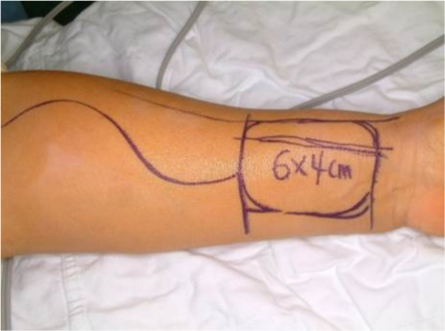
During operation (patient no. 7)

**Fig. 16:**
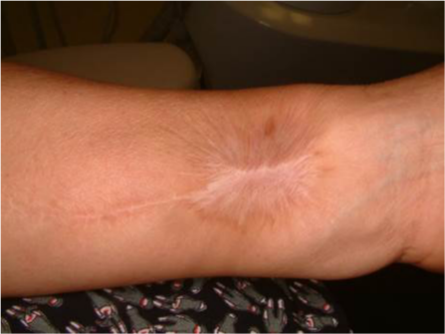
Postoperation 2 years 10 months (patient no. 7)

**Fig. 17:**
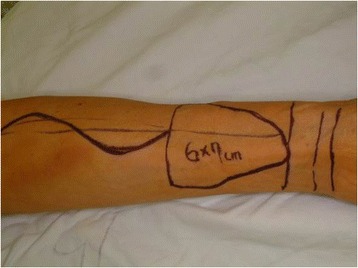
Tendon exposure case: during operation

**Fig. 18:**
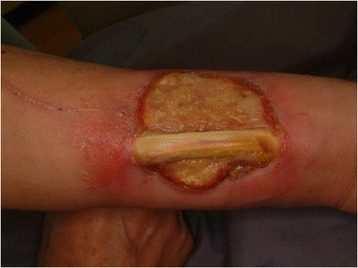
Tendon exposure case: tendon exposure after 2 months postoperatively

**Fig. 19:**
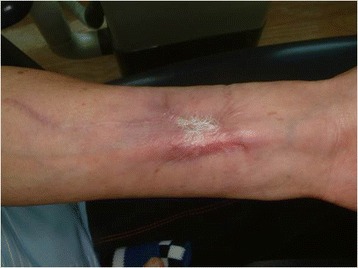
Tendon exposure case: healing after 11 months postoperatively

Donor site complications including graft rejection, graft failure, infection, impaired hand manipulation, and swelling of hand were not noted in any patient. There was no functional defect or impairment 3 months after operation. One patient had suffered from tendon exposure at follow-up visit 2 months from surgery. The other 21 patients were satisfied with the esthetic results. Three patients died of recurred buccal mucosa cancer 17, 22, and 30 months after operation, respectively (patient nos. 2, 13, and 15 in Table 1). During the healing period, epithelial migration was over the artificial dermis graft from the adjacent normal skin toward the center of the donor site defect. The original donor defect were ranged from 20 to 48 cm^2^, and the mean defect size was 31 cm^2^ (Table 1). The final scar sizes were ranged from 3.3 to 19.8 cm^2^ except one case of tendon exposure patient. The mean of final scar size was 11.6 cm^2^. The original donor defect of the tendon exposure case was 42 cm^2^, but 2 months later, the scar size was measured to 40.5 cm^2^ approximately. On average, the final scar area was decreased to 36.1 % size in average compared to the original donor defects. The complete healing was obtained between 1.5 and 3 months according to the defect size in 21 patients (Figs. 5, 6, 7, 8, 9, 10, 11, 12, 13, 14, 15, and 16). The scar areas of the most patients were not changed markedly and remained stable after the 3 months from the surgery. Tendon exposure was observed in one patient in 22 patients. He suffered some donor site discomfort functionally and esthetically. Prolonged treatment including periodical follow-up and wound dressing was done until complete wound healing was conformed after 11 months postoperatively (Figs. 17, 18, and 19).

**Table 1:** Comparison between donor site and cicatrical area

Patient no.	Sex	Age	Diagnosis	Donor site area (cm^2^)	Cicatrical area (cm^2^)	C/D ratio
1	F	65	SCC	25	6.9	0.28
2	F	84	SCC	48	13.2	0.28
3	M	67	SCC	24	4.2	0.18
4	M	27	SCC	20	12.6	0.63
5	F	66	SCC	30	19.8	0.66
6	F	73	Melanoma	48	10.5	0.22
7	F	64	SCC	24	3.3	0.14
8	M	71	SCC	28	7.9	0.28
9	F	65	SCC	24	3.3	0.14
10	M	66	SCC	20	6.1	0.31
11	M	20	SCC	42	18	0.43
12	M	61	SCC	20	6.4	0.32
13	M	39	SCC	30	18.3	0.61
14	M	54	SCC	40	11.5	0.29
15	M	58	SCC	25	6.6	0.26
16	F	75	SCC	24	3.3	0.14
17	M	61	SCC	40	17	0.43
18	F	72	SCC	24	3.2	0.13
19	F	75	SCC	20	5.85	0.29
20	M	61	SCC	36	16.2	0.45
21	M	70	SCC	42	40.2	0.96
22	F	48	SCC	48	12.2	0.25
Average		61		31	11.2	

(*C/D* cicatrical area/donor site area, *F* female, *M* male, *SCC* squamous cell carcinoma)

## Discussion

Many investigators have reported that closing donor sites with FTSG had resulted satisfactory outcomes functionally and esthetically [11]. But, FTSGs from distant sites have the disadvantages of requiring graft harvesting, causing relatively large donor site scars, and the potential need of STSG or other substitutes to close FTSG donor sites [5]. In this respect, STSG can be a reasonable alternative to FTSG. But, even with normal healing of the STSG at the radial forearm donor site, the result may cause poor esthetic results with color and contour irregularities [5]. In addition, the STSG has much less volume than FTSG and lacks connective tissue for successful reunion to donor site; therefore, STSG is more susceptible to trauma and late complications, including contracture of the graft and exposure of structures under the skin. For such reasons, several defiant attempts had been suggested including artificial dermal materials with STSGs. Wester et al. suggested that the use of artificial dermis with STSG could have provided thicker coverage of the forearm defect, with minimal donor site morbidity and superior cosmetic results compared with STSG alone [9]. Compared to the use of STSG alone, the use of an artificial dermis that contains elastin in combination with a STSG can lead to minimized contracture and enhanced skin elasticity, so is very effective functionally and esthetically [12]. The combination of proper artificial dermis and a STSG can be a reasonable alternative to minimize donor site morbidity.

In this study, we tried to replace epidermis with silicon-contained bilayer artificial dermis. We used MatriDerm of inner layer and Terudermis of outer layer for the coverage of the forearm donor site. Terudermis has a bilayer structure consisting of an outer silicone layer and an inner layer of mixed atelocollagens [13]. The inner collagen layer of Terudermis acts as a tissue scaffold for migration and ingrowth of dermal cells. Therefore, this layer can minimize contraction and scarring of full-thickness skin defect. Meanwhile, the outer silicon layer functions as a barrier for protection against foreign body infiltration into wound site. Terudermis is a bilayer graft for dermal defects consisted of collagen and silicon membrane. Inner layer consisted of fibrillary atelocollagen and heat-denatured atelocollagen, which promotes cell migration. And, an outer silicon membrane about 100 μm thick is bound to the collagen layer forming a bilayer membrane [13]. This new bilayer artificial dermis, formed by dehydrothermal crosslinking, has biocompatible and stable properties [14]. Fujioka and his colleagues suggested that the efficacy of these artificial skin substitutes included to treat full-thickness skin defects as well as improve the quality of the skin [15]. Atelocollagen sponges have been applied to cover full-thickness skin defects as an artificial dermis [16]. As an artificial dermis containing atelocollagen sponges, Terudermis was approved for clinical use in the treatment of the injured dermis and epidermis by the Ministry of Health in Japan, and has been proven to improve skin regeneration of dermal or mucosal injuries without any critical adverse effects [17]. The use of artificial dermis in radial forearm flap donor closure may need an additional procedure of secondary STSG. But, using silicon-contained bilayer artificial dermis like Terudermis can be a substitution of STSG for a certain period of time.

MatriDerm, which was made commercially available in 2004, minimizes contracture of scar and increases hand and wrist movement [18]. MatriDerm consisted of various types of collagen, and it provides a scaffold to reconstruct the skin and reduces scar formation [7, 19]. Choi et al. suggested that the simultaneous application of MatriDerm and the STSG can lead functional and esthetical improvement and therefore it plays the role of a temporary barrier with the various advantages including the maintenance of the elasticity and flexibility [18]. For a dermal matrix, too short period of degradation in the wound environment is unfavorable for minimizing donor site complications [20]. The crosslinking structure of MatriDerm makes it possible to heal during the long period of 3–4 weeks for vascularization of artificial dermis [20]. MatriDerm is composed of insoluble collagen fibers, which are crosslinked to elastin, and these collagen materials can remain in perfection until the secondary healing stage [21]. During healing period, the collagen fibers were from a scaffold that induces fibroblasts and other cells to regenerate dermal structure [21]. Elastin included in MatriDerm plays a role of reducing wound contraction [7, 20]. But, when using MatriDerm, a drawback is the cost of 4.5US$/cm^2^. Therefore in our cases, the costs for the substitution ranged between 90 and 216US$ in the USA [21]. But, in South Korea, MatriDerm is an imported product, so the cost of MatriDerm is much higher than the cost in the USA. Terudermis is also an imported product and is one of expensive biomaterials without the subsidization by national health care insurance in South Korea.

In our study, without the second donor site morbidity caused by elevation of split-thickness skin, we evaluated the effectiveness of this technique in consideration of decreased scar sizes and patient’s comfort during healing period. It is a major advantage of this procedure to skip of secondary surgery for donor site coverage. The other disadvantage of using artificial dermis without STSG is a delayed healing period. The healing period of an STSG is typically 4 to 6 weeks [22]. In the condition of using artificial dermis only, the healing accomplishes secondarily and the period may be prolonged for over 3 months. It may help to reduce the exposure of infra-dermal structures like tendon. Davis et al. evaluated the outcomes between FTSG versus STSG for coverage of RFFF donor sites [8]. In their study, there was no significant difference between two groups, and tendon exposures were founded in 16.1 % of the STSG group and 31.3 % of the FTSG group [8]. In other study of donor site evaluation, Ito et al. covered the donor site by FTSG from the groin. They said that the complications including depression and wrist mobility limitation were acceptable, but the scar size of radial forearm donor site was rather increased in 21.7 % of patients [11]. In our study, tendon exposure was observed in one patient. Within only a few weeks postoperatively, he had to resume the work which needed a lot of manual labors. He underwent tendon exposure and discomfort of hand movement but after additional periodical checkups and wound dressings, exposure tendon was covered by newly formed skin eventually.

## Conclusions

Double-layered collagen graft to the donor site of the forearm flap was successfully performed in this study. Patients were satisfied with the esthetic and functional results. Without the thigh skin graft, patients had experienced less painful postoperative healing periods and discomfort. Therefore, double-layered collagen graft without STSG represents a viable option for radial forearm donor site reconstruction.
